# The feasibility of a single-blinded fast-track pragmatic randomised controlled trial of a complex intervention for breathlessness in advanced disease

**DOI:** 10.1186/1472-684X-8-9

**Published:** 2009-07-07

**Authors:** Morag C Farquhar, Irene J Higginson, Petrea Fagan, Sara Booth

**Affiliations:** 1 General Practice and Primary Care Research Unit, Dept Public Health & Primary Care, University of Cambridge, Institute of Public Health, Robinson Way, Cambridge, CB2 0SR, UK; 2 Department of Palliative Care, Policy & Rehabilitation, King's College London, Weston Education Centre, Cutcombe Rd, London, SE5 9RJ, UK; 3 Breathlessness Intervention Service, Box 193, Addenbrooke's Hospital, Cambridge University Hospitals' NHS Foundation Trust, Hills Road, Cambridge, CB2 0QQ, UK

## Abstract

**Background:**

The Breathlessness Intervention Service is a novel service for patients with intractable breathlessness regardless of aetiology. It is being evaluated using the Medical Research Council's framework for the evaluation of complex interventions. This paper describes the feasibility results of Phase II: a single-blinded fast-track pragmatic randomised controlled trial.

**Methods:**

A single-blinded fast-track pragmatic randomised controlled trial was conducted for patients with chronic obstructive pulmonary disease referred to the service. Patients were randomised to either receive the intervention immediately for an eight-week period, or receive the intervention after an eight-week period on a waiting list during which time they received standard care. Outcomes examined included: response rates to the trial; response rates to the individual questionnaires and items; comments relating to the trial functioning made during interviews with patients, carers, referrers and service providers; and, researcher fieldwork notes.

**Results:**

16 of the 20 eligible patients agreed to participate in a recruitment visit (16/20); 14 respondents went on to complete a recruitment visit/baseline interview. The majority of those who completed a recruitment visit/baseline interview completed the RCT protocol (13/14); 12 of their carers were recruited and completed the protocol. An unblinding rate of 6/25 respondents (patients and carers) was identified. Missing data were minimal and only one patient was lost to follow up. The fast-track trial methodology proved feasible and acceptable. Two of the baseline/outcome measures proved unsuitable: the WHO performance scale and the Schedule for the Evaluation of Individual Quality of Life-Direct Weighting (SEIQoL-DW).

**Conclusion:**

This study adds to the evidence that fast-track randomised controlled trials are feasible and acceptable in evaluations of palliative care interventions for patients with non-malignant conditions. Reasonable response rates and low attrition rates were achieved. Further, with adequate preparation of the research and randomisation teams, clinicians, and responders, and effective liaison with the clinicians, single-blinding proved possible. Methods were identified to reduce unblinding through careful attention to the type of data collected at unblinded measurement points; the content of interviews should be carefully considered when designing blinded-trial protocols.

**Trial registration:**

Clinical Trials.gov NCT00711438

## Background

Intractable breathlessness is common in advanced disease, both malignant and non-malignant. In chronic obstructive pulmonary disease (COPD) and heart failure it is nearly universal by the time of death, its prevalence reaching 90–95% and 60–88% respectively in the advanced stages [1]. Recent advances in the palliation of breathlessness include non-pharmacological intervention services to reduce or contain the severity of the sensation. Formally evaluated services have had positive outcomes in terms of reduction in distress caused by breathlessness, increased functioning and quality of life [2-5], however these services focused solely on patients with malignant disease and their evaluations were methodologically limited (e.g. only two were randomised controlled trials (RCTs) and published outcomes were limited to patient outcomes only). These were complex interventions which are notoriously difficult to evaluate [6].

The Medical Research Council's Framework for the Development and Evaluation of RCT for Complex Interventions to Improve Health [6] was developed to combat the unique challenge of evaluating complex interventions by building a continuum of increasing evidence to support the effective development of complex interventions through robust methodology. It was described as the current method of choice for assessing complex interventions for breathlessness by the 2005 Medical Research Council (MRC) Clinical Trials Unit/Cicely Saunders Foundation 'Improving Research Methodology in Breathlessness' meeting [7].

The Breathlessness Intervention Service (BIS) Service at Addenbrooke's NHS Trust is being evaluated using the MRC framework [6]. BIS aims to manage the symptom of intractable breathlessness in patients with disease of any aetiology (malignant and non-malignant) using a rehabilitative approach. Interventions include evidence-based non-pharmacological interventions (psychological, social and physical), palliative care input (e.g. end of life issues, psychosocial issues, family concerns) and pharmacological review. Thus BIS seeks to enhance the self-management of breathlessness. Uniquely, care is flexibly located preferentially in patients' own homes, or in a clinic if more appropriate. Referrals come from medical specialists, GPs and allied health professionals (with medical consent). At Phase II BIS was staffed by a clinical specialist physiotherapist and palliative care consultant.

The MRC framework has recommended RCTs as the gold standard for evaluating services [6], however conducting RCTs within palliative care is challenging because of difficulties with recruitment, due to acceptability, and attrition, associated with increasing morbidity and death [8]. Following the experience of Higginson et al (2006 & 2008) [9,10], a fast-track RCT model was selected to address these difficulties. This design has previously been attempted in palliative care by McWhinney et al (1994) [11] to evaluate a palliative care home support team but this single-blinded trial failed to recruit an adequate sample size due to problems with attrition and inaccurate prognostication; it is unclear what diagnoses the intervention was targeted at, but it was likely to have been patients with malignancies. The recent successful palliative care fast-track RCT by Higginson et al (2006 & 2008) evaluated a palliative care service for people affected by multiple sclerosis, however the authors made no attempt at blinding [9,10]. Hart et al (2008) describe the 'impossibility' of double-blinding in experience-based trials, but stress the importance of single-blinding the investigator where possible [12]. Thus the Phase II study reported here sought to test the feasibility of single-blinding in a fast-track pragmatic RCT of BIS versus standard care for patients with a different non-malignant disease (COPD) and their informal carers. Results of the earlier phases of the development and evaluation of BIS have been published elsewhere [13,14].

## Method

### Sample

Although the BIS accepts referrals for patients with both malignant and non-malignant conditions, COPD patients make up the largest diagnostic group of referrals. In addition, a body of work (cited earlier) has begun to address the role of such interventions (albeit only in a clinic setting) in patients with malignancies. Therefore this Phase II trial focused on a homogeneous cohort of COPD patients, although BIS continued to deliver the intervention to patients with other diagnoses such as cancer and heart failure outside of the trial.

### RCT design

A single-blinded fast-track pragmatic RCT design was used. Patients were randomised either to a fast-track group (FT), where they received the intervention immediately, or the control condition (the waiting list group; WL), where they received the intervention after an eight-week period on a waiting list during which time they received standard care.

### Definition of intervention and standard care

Table 1 defines the intervention that BIS provided, in terms of a minimum set of core interventions for non-malignant patients at Phase II.

**Table 1:** Service model for the Breathlessness Intervention Service (BIS) for non-malignant patients at Phase II RCT (model date: 15/12/06: since revised)

Target patient group:	Refractory dyspnoea – chronic breathlessness which is medically optimally managed
Referral:	Post, fax, electronic

Assessment lead:	Clinical Specialist Physiotherapist

BIS team:	❑ Clinical Specialist Physiotherapist: expert in three different disease groups (cancer, heart failure, COPD), conducts highly specialised assessment, works off-site and on-site.
	❑ Palliative Medical Consultant

Medical assessment:	May be required

Average no. of home visits:	3

Average no. of telephone contacts:	3

Ratio of face-to-face to telephone:	1:1

Average length of service contact:	6–8 weeks

Outcome measures collected at first assessment:	❑ modified Borg [19] at rest, self-reported, on exertion completion of exercise test
	❑ anxiety due to breathlessness at rest, self reported, on exertion & on completion of exercise test
	❑ physiological measures e.g. oxygen saturation, heart rate & respiratory rate

Non-pharmacological interventions:	1^st ^stage of intervention

Pharmacological interventions:	2^nd ^stage of intervention

1^st ^stage interventions (selection & application as clinically indicated, majority used):	❑ explanation & reassurance
	❑ anxiety management
	❑ psychological support
	❑ hand-held fan
	❑ information fact sheets
	❑ emergency plan
	❑ positioning to reduce work of breathing (rest, recovery & activity)
	❑ breathing control
	❑ education to patient, carer & health care generalists
	❑ pacing & lifestyle adjustment
	❑ individualised exercise plan
	❑ relaxation & visualisation
	❑ airway clearance techniques
	❑ advice regarding nutrition & hydration
	❑ support to family & patient to utilise education & self-support programmes
	❑ sleep hygiene
	❑ smoking cessation prompt
	❑ brief cognitive therapy
	❑ pharmacological review

2^nd ^stage interventions (choice dependent on outcome of first stage interventions):	❑ further pharmacological review e.g. low dose opioids, anti-depressants, anxiolytics
	❑ referral to specialist services (see below)
	❑ referral for long term oxygen therapy (LTOT) or short burst oxygen therapy (SBOT) assessment

Other symptom management:	May be required

Documentation:	❑ individualised patient plan
	❑ discharge summary to referrer with copies to GP, specialist services the patient was already in contact with (e.g. respiratory physicians), other involved health care professionals (e.g. district nurses, nursing home care staff)

Referrals:	❑ Pulmonary rehabilitation
	❑ Specialist dietetic
	❑ OT advice
	❑ Specialist psychological services
	❑ Hospice day services
	❑ other specialist assessment
	❑ (n.b. these services usually have a wait time)

The control group received standard care for an eight-week period before receiving BIS. Our definition of standard care, in the context of this Phase II RCT, was: specialist outpatient appointments in secondary care (e.g. respiratory) that may include specialist nurse input, and primary care services.

### Single-blinding

Various placebos were considered but found to be unworkable for the clinicians and unbelievable as 'interventions' to the patient and carer populations. It was also considered possibly unethical for the clinicians given that BIS was using evidence-based interventions. Further, it was impossible to blind the clinicians to the group to which patients were allocated as the providers delivered the intervention to both groups and had necessary access to their referral histories (i.e. dates of referral). In pragmatic trials of services, blinding patients and clinicians is nearly always impossible [8,15]. For this Phase II trial an attempt was made to blind the researcher to the allocation of respondents until the end of the intervention period (week 8).

This was achieved by the researcher conducting the recruitment to the study and collecting baseline measures, but then passing the process of randomisation and reporting of allocation over to a third party (Addenbrooke's clinical trials' team). Patients were randomised to the fast-track group (FT) or the waiting list group (WL) by the clinical trials' team sequentially opening sealed opaque numbered envelopes containing the random group allocation previously generated by a computer programme at King's College London (KCL). The envelopes were set up by an administrator at KCL not associated with the study.

Patients were then informed of the outcome of randomisation by the clinical trials' team, by telephone. BIS was then notified of the outcome of randomisation by the clinical trials' team (by telephone and secure email) in order that the service could book the first appointment with the patient in-line with the study protocol. The researcher was then notified that randomisation had occurred, and that the patient and service had been informed, but not the outcome of the randomisation. The purpose and need for single blinding was explained to patients and carers at the recruitment visit and they were reminded at the start of each subsequent blinded interview to try not to let the researcher know their group allocation. In addition, all data were handled using study identity numbers; group allocation identifiers were only added at the analysis stage.

Data collected from the waiting list group once they were in receipt of BIS (after their period on the waiting list when their group allocation was blinded to the researcher) was treated as before/after data and not RCT data. This allowed for the collection of qualitative data at the midpoint of using the service for the waiting list group as the researcher was no longer blinded to their group allocation, nor was she required to be at this stage.

### Measurement points

**Figure 1:**
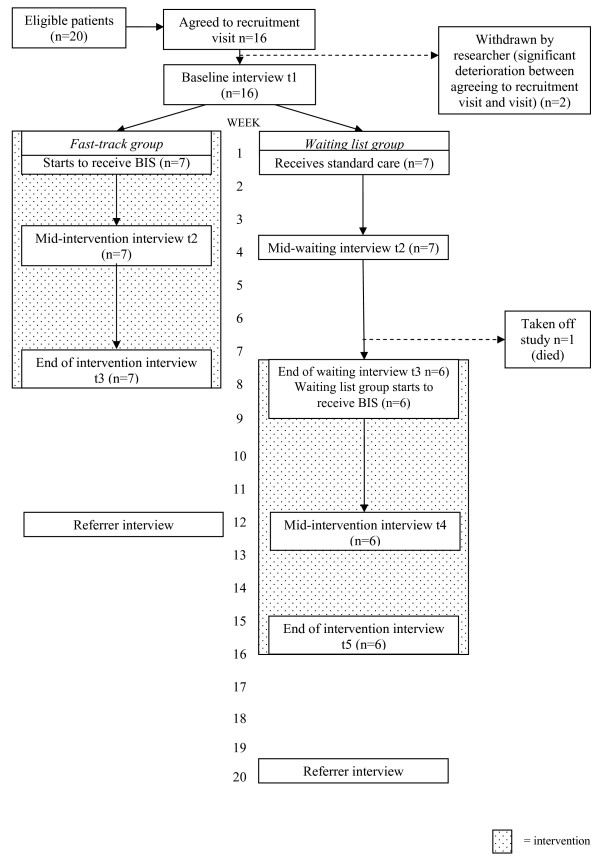
Flow chart depicting the follow up measurement points

Quantitative and qualitative data were collected by interview for all respondents at baseline (t1), prior to randomisation. A flow chart depicting the follow up measurement points is given in Figure 1.

### Sample size

As this was a feasibility study, comparative analysis was not our primary objective thus adequate powering of the trial was not required. Based on clinical and research experience and data published from other studies we aimed to recruit a maximum of 28 patients to the trial. Phase II would then provide the data, at this time lacking, to inform the power calculations for a Phase III RCT.

### Baseline and outcome measures

Baseline characteristics and outcomes included patients' breathlessness, patient and carer distress due to breathlessness, patient mastery of breathlessness, patient and carer quality of life, patient and carer anxiety, other service use, caregiver burden, and patient and carer expectations of and satisfaction with the service. Table 2 outlines the baseline and outcome measures selected following a review of the literature [7,16-22]. The resulting interview schedule was both quantitative and qualitative at each measurement point except t2; thus this was a mixed methods RCT.

**Table 2:** Patient and carer baseline and outcome measures for BIS Phase II RCT

**Baseline characteristic/outcome**	**Instrument/measure**
**PATIENT**	

Patient breathlessness	Modified Borg [19] at rest and on exertion & identification of activity that makes breathlessness worst

Patient breathlessness at best/worst	Visual Analogue Scale (VAS) [7]

Patient functional ability	WHO performance scale [16]

Patient social functioning	No. of times patient goes out of the house (average for last month)

Patient quality of life	SEIQoL-DW [17]

Patient satisfaction with the service	Qualitative (t1 – expectations of the BIS; t3/t5 – useful aspects of the service)

Patient critical incidents data	Qualitative (e.g. identification of examples of difficulty getting help/medication/advice needed)

Patient anxiety	Hospital Anxiety and Depression Scale [20]

Patient distress due to breathlessness	Visual Analogue Scale for distress caused by breathlessness [7]

Patient mastery of breathlessness	Mastery items from Chronic Respiratory Questionnaire [18]^1^

Patient use of other services	Service use questions

**CARER**	

Carer quality of life	SEIQoL-DW [17]

Carer satisfaction with the service	Qualitative (t1 – expectations of the BIS; t3/t5 – useful aspects of the service)

Carer critical incidents data	Qualitative (e.g. identification of examples of difficulty getting help/medication/advice needed)

Carer anxiety	Hospital Anxiety and Depression Scale [20]

Carer's assessment of patient's breathlessness	Modified Borg [19] at rest and on exertion & identification of activity that makes breathlessness worst

Carer's assessment of patient's breathlessness at best/worst	VAS adapted for carer

Carer distress due to patient's breathlessness	VAS adapted for carer – distress caused to carer by patient's breathlessness

Carer's assessment of patient's use of services	Service use questions

Carer's social functioning	No. of times carer goes out of the house (average for last month)

Caregiver burden	Burden Interview [21] Caregiver Appraisal Scale [22]

^1^Confirmation that it was valid to use these items in isolation of the whole Chronic Respiratory Questionnaire was received from its developer, Gordon Guyatt (email communication, 2005).

Despite not powering the trial, a primary outcome measure was identified in order that the results could inform the sample size calculations for Phase III. The primary outcome measure was 'distress due to breathlessness' measured using a Visual Analogue Scale (VAS; 0–10 cm); a difference between the baseline and follow up measurements of 1 cm on this scale could be regarded as clinically significant for patients with intractable breathlessness [23].

### Inclusion and exclusion criteria

Table 3 shows the inclusion and exclusion criteria. These criteria were established in order to ensure the representativeness of the study sample to the population who would receive the BIS outside of the trial.

**Table 3:** Inclusion & Exclusion criteria for entry to BIS Phase II RCT

**Inclusion criteria**	
Patients	i) Appropriate referral to the BIS
	ii) Diagnosis of COPD/COAD
	iii) Aged 18 years or over
	iv) Any patient who does not meet any of the exclusion criteria

Carers	i) The informal carers of patients specified above, who can be significant others, relatives, friends or neighbours
	ii) Aged 18 years or over
	iii) Any carer who does not meet the exclusion criteria

**Exclusion criteria**	

Patients/carers	i) Any patient/carer unable to give informed consent
	ii) Any patient living outside of Cambridgeshire PCT, West Essex PCT, East & North Hertfordshire PCT, or Suffolk PCT
	iii) Any patient who has previously had access to BIS
	iv) Any patient/carer who is demented or confused
	v) Any patient/carer with learning difficulties
	vi) Any patient/carer from other vulnerable groups (e.g. head injury, severe trauma, and mental illness)

### Data collection

Recruitment, randomisation and baseline interviews were conducted over a ten-month period between late March 2006 and early January 2007; the final patient interview was conducted in March 2007. All measurement points were audio-taped with respondents' permission and were conducted by experienced researchers (MF and GE). All patient interviews were conducted in their own homes; interviews with carers were conducted in their own homes, at their place of work or at their patient's home according to preference. Wherever possible, patients and carers were interviewed separately. Careful attention was paid to need, in terms of fatigue.

### Data management

Quantitative data (e.g. structured questionnaires) were entered into SPSS. Interviews t1, t3 and t5 were transcribed by an independent transcription company, checked for accuracy and anonymised. Transcripts were then imported into NUD*IST software to enable storage and organisation of the data for analysis.

### Approvals and trial registration

Ethics approval was obtained from the Cambridge LREC and R&D approval from Addenbrooke's R&D and the relevant local Primary Care Trusts (REC reference no. 05/Q0108/471). The trial was registered with ClinicalTrials.gov (NCT00711438).

### Data analysis

In order to examine the feasibility of the trial methodology, the following were examined: response rates to the trial; response rates to the individual questionnaires and items; comments relating to the trial functioning made during interviews with patients, carers, referrers and BIS providers; and, researcher fieldwork notes.

## Results

Our original sample size was 28 patients. As the Phase II exploratory RCT progressed it became clear that: 1) BIS for patients with COPD could be conducted over a shorter time period than eight-weeks; 2) BIS needed to be evaluated across the full disease spectrum it served (malignant as well as non-malignant diseases other than COPD); 3) the service model for BIS was different for malignant and non-malignant conditions due to the differing disease trajectories, such that separate RCT protocols for evaluation of BIS for malignant and non-malignant conditions would be required; 4) some of the outcome measures were unsuitable or could be improved upon; 5) there would be difficulty maintaining clinical equipoise for COPD patients for a Phase III definitive RCT if the exploratory RCT continued; 6) assessment of cost effectiveness was warranted. Thus Phase II was discontinued 10 months into the RCT. Further details on these points follow in this and the following discussion sections.

At the time of discontinuation, 20 patients had met the inclusion criteria for Phase II. No patients with COPD referred to BIS during the study period were excluded. Table 4 shows the response rates of the 20 eligible patients.

**Table 4:** BIS Phase II RCT response rates

**ID No.**	**Agreed to recruitment/baseline visit?**	**Recruitment visit completed?**	**Reason for non-response**
001	Yes	Yes	

002	No	n/a	Moving house

003	Yes	Yes	

004	Yes	Yes	

005	Yes	Yes	

006	Yes	Yes	

007	Yes	No – withdrawn by researcher & referred straight to BIS	Significant deterioration between agreeing & baseline interview (t1)

008	No	n/a	Admitted to ITU – too ill on discharge

009	Yes	Yes	

010	No	n/a	None given

011	Yes	Yes	

012	No	n/a	Too ill (nursing home resident)

013	Yes	Yes	

014	Yes	Yes (but carer unobtainable)	

015	Yes (but died on waiting list; carer refused)	Yes (but died on waiting list; carer refused)	

016	Yes	Yes	

017	Yes	Yes	

018	Yes	No – withdrawn by researcher & referred straight to BIS	Admission & significant deterioration between agreeing & baseline interview (t1)

019	Yes	Yes	

020	Yes	Yes	

Sixteen of the 20 patients invited to participate agreed to a recruitment visit, 14 completed the recruitment visit and 13 went on to complete the RCT protocol. Two patients were withdrawn by the researcher between agreeing to the recruitment visit and the conduct of the recruitment visit (one following research-carer phone discussions between the two events, and one on arrival of the researcher at the recruitment visit). This occurred prior to informed consenting and randomisation, and was due to significant deterioration in the patients' health: one was admitted to hospital with an acute exacerbation and the other was clearly entering the terminal stage and would not have been able to participate (the carer had called the GP just prior to the researcher's arrival).

No difficulties were reported by the third party randomisation team (Addenbrooke's Clinical Trials Team) either in terms of managing the randomisation process, contacting patients, contacting BIS or contacting the researcher. Table 5 shows the outcome of randomisation.

**Table 5:** BIS Phase II RCT randomisation outcome

**Recruitment/randomisation order**	**Recruitment/ID no.**	**Fast-Track group**	**Waiting List group**	**Unblinded early?**
1	01	1		By patient at t2

2	03		1	No

3	05	2		No

4	04		2	By patient

5	06		3	By patient at t3 (early)

6	09	3		No

7	11		4	No

8	13	4		No

9	15 (died pre t3)		(X)	No

10	14	5		By patient on phone pre t3

11	16	6		By patient at t2

12	17		5	By carer at t1 (conducted post-patient's t1 and randomisation) *

13	19		6	No

14	20	7		No

Total		7	6	6/13

* unblinded to wrong group

**Figure 2:**
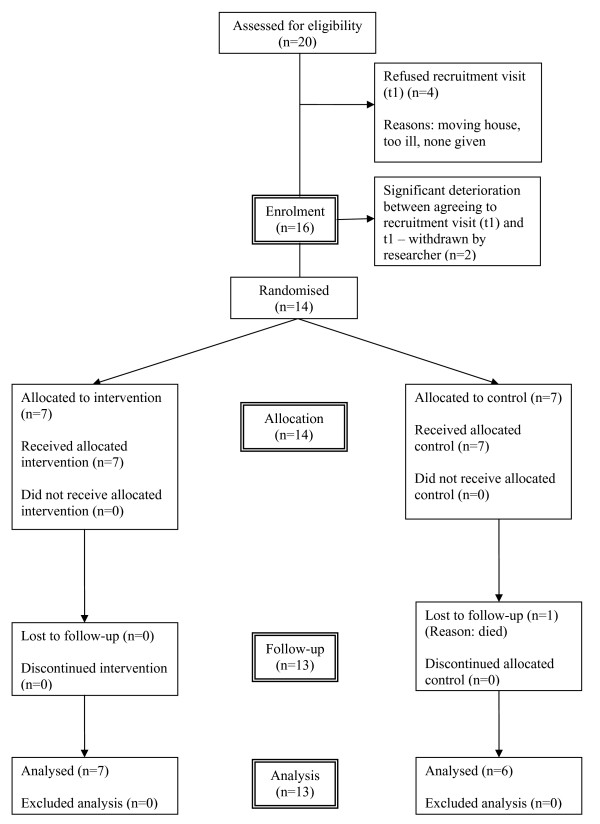
CONSORT flow chart summarising the numbers for study enrolment, randomisation, allocation, follow up and analysis

Seven patients were randomised to the fast-track (FT) group and seven to the waiting list (WL) group. One patient died whilst on the waiting list so reducing the final WL sample size to six: this was the only patient lost to attrition. Figure 2 provides a CONSORT flow chart summarising the numbers for study enrolment, randomisation, allocation, follow up and analysis. Carers were identified for all 13 respondents however one could not be contacted due to a house move; 12 therefore participated and completed the carers' protocol.

As described earlier, the protocol was designed in order that the researcher could remain blinded to group allocation until the qualitative questions at t3 (week 8). In this pragmatic trial five of the 13 patients and one of the 12 carers who completed the study unblinded the researcher prior to this point (see Table 5). One of these unblindings (a carer) was to the wrong group i.e. the carer implied that their patient was in the fast-track group (receiving BIS) when in fact they were in the waiting list group and the patient had not yet seen BIS. Including this latter unblinding, there was therefore a 6 out of 25 respondents unblinding rate (base number consists of 13 patients and 12 carers). All unblindings appeared to be accidental rather than deliberate, and unblinders did not appear to be aware that they had unblinded themselves. Most unblindings took the form of mentioning a visit or contact by the BIS physiotherapist.

### Sample characteristics

The age range of responding patients was 53–80 years, with a median of 69 years. The majority of patients were male (8/13). Their most recent FEV1 ranged from 0.68–1.28 litres/min and % predicted ranged from 12.6–28.9%. The age range of responding carers was 29–76 years, with a median of 57 years, and the majority of carers were female (9/12).

### Patient and carer missing data

For each patient and carer variable and outcome variable results are given in terms of the feasibility findings (missing data and comments on acceptability) for patients and for carers. Missed measurement points for patients and carers were minimal. As noted above, one carer could not be contacted at all due to a house move and one patient died before t3 and was therefore lost to attrition. The obtained and missing data points for the remaining 13 patients and 12 carers are given in Table 6.

**Table 6:** Obtained and missing data measurement points for patients and carers in BIS Phase II RCT

**ID No.**	**Interview no.**									
	
	**t1**		**t2**		**t3**		**t4**		**t5**	
	
	**Pt**	**Carer**	**Pt**	**Carer**	**Pt**	**Carer**	**Pt**	**Carer**	**Pt**	**Carer**
001	D	D	D	D	D	D	n/a	n/a	n/a	n/a

003	D	D	D	D	D	D	D	D	D	D

004	D	D	D	D	D	D	DI	DI	D	D

005	D	D	D	D	D	D	n/a	n/a	n/a	n/a

006	D	D	D	D	D	D	D	DP	D	D

009	D	D	D	D	D	D	n/a	n/a	n/a	n/a

011	D	D	D	D	D	D	D	D	D	D

013	D	D	D	D	D	D	n/a	n/a	n/a	n/a

014	D	U	D	U	D	U	n/a	n/a	n/a	n/a

016	D	D	D	D	D	D	n/a	n/a	n/a	n/a

017	D	D	D	D	D	D	DP	DP	D	D

019	D	D	D	X	D	DP	DP	DP	D	D

020	D	D	D	D	D	D	n/a	n/a	n/a	n/a

No. taped	13	12	13	11	13	11	3	2	6	6

No. of interviews/total sought	13/13	12/13	13/13	11/13	13/13	12/13	6/6	6/6	6/6	6/6

Key:

D = completed data collection point by interview and taped

DI = completed data collection point by interview, but not taped

DP = completed data collection point postally (at patient/carer request due to holiday), but not taped

n/a = not applicable i.e. in fast-track group

X = data collection point missed due to carer burden (family illness and other caring commitments)

U = carer unavailable due to house move

For those completing the protocol Table 6 shows that there were no missed patient measurement points and only one missed carer measurement point: this was for a t2 interview for a carer whose caring role to the trial patient was secondary to another, primary, caring role: this measurement point was abandoned due to acute illness in the carer's family.

Quantitative patient and carer missing data for individual questionnaires and items is reported in Table 7 (Additional File 1).

### Trial methodology

The fast-track trial methodology proved acceptable to the patients, carers, referrers and BIS providers. No complaints or negative comments were received from any participants randomised to the waiting list group about their period of waiting. The timings of the interviews (measurement points) were manageable from a researcher perspective, fitted with the service provision and appeared acceptable to respondents.

Patient and carer baseline and final interviews lasted between an hour and an hour and a half (approximately); other follow up interviews lasted between 30 minutes and an hour (approximately). Patients were contacted by telephone during the morning of each planned interview before the researcher set out, to re-check the suitability of the appointment in terms of the patients' health status that day: this resulted in a small number of interviews being re-scheduled.

### Service model

In addition to these findings relating to the feasibility of the trial there were findings relating to the feasibility of the service model collected through interviews with the BIS staff themselves. The service model for Phase II was an eight-week intervention however it became clear that a more focused intervention could be conducted over a four-week period. This would have the added advantage of reducing the waiting time for patients randomised to the waiting list group to four-weeks in Phase III, so potentially increasing acceptability still further.

## Discussion

### Response rate

Although based on a small sample size, this Phase II RCT achieved a reasonably good response rate for a trial conducted in palliative care that is worthy of further comment. Possible reasons for this response rate include the relationship between the research and clinical team, the patient recruitment letter, the use of a fast-track design, the patient group, and patient altruism. The relationship between the research and clinical team was undeniably strengthened by the research-culture of the clinical team, in particular its desire to learn from the various phases of the evaluation and remodel the service accordingly. The evaluation was initiated by the lead clinician who founded the service (SB) and who invited in academic collaboration. The service has been involved in every phase of the evaluation but with all aspects of data collection and much of the analysis being conducted independently of it. This is similar to the participatory approach described by Hopkinson et al (2005) [24]. The frequently reported gate-keeping role of service providers [25-28] or lack of support from clinical colleagues [8] was therefore not an issue for this study.

A notable feature of the patient recruitment letter was that, at the ethics committee's insistence, it was printed on BIS's own clinical letterhead (which indicated it was part of the palliative care team), as opposed to on the academic letterhead of the independent research team (i.e. King's College London). This may have increased patients' acceptance of the trial.

Palliative care patients, and their families, can be reluctant to participate in trials with a traditional design, where patients are randomised to an intervention or no intervention [8]. The use of a fast-track design meant that all patients had access to the intervention and, in addition, those in the fast-track group had access to it earlier than would normally be the case. Within the context of NHS culture patients are familiar with waiting lists and often expect to wait for a service: thus the short lead-in time to receiving BIS for those randomised to the fast-track group may have increased the response rate to this study.

Further the patient group targeted by this study may in itself have had an impact on study recruitment. Services for COPD patients are inconsistent and sporadic [13,29-31], and access to palliative care is limited [32-34]. Thus access to any service for these patients is likely to be desirable, and a novel service particularly so. This may have been further compounded by an appreciation of research focusing on a symptom that is distressing, yet not often discussed openly, as highlighted in the earlier phases of this research [13,14,35]. One Phase II patient described the symptom of breathlessness, unlike pain, as 'not very fashionable' (P006).

### Low attrition & missing data rates

The study achieved a low attrition rate and low missing data rate, again unusual for a palliative care RCT [8,10]. Using the same fast-track design Higginson et al (2008) [10] had lower still attrition and missing data rates: they suggested that the disease trajectory of the target group, combined with the use of a fast-track design and highly skilled interviewers were the likely explanations for their success; the same reasons are likely to hold true for this study. A similar study with patients with malignant disease may not achieve such relatively low rates. The small sample size negates the possibility of exploring the relationship between patient characteristics and the response rates, attrition or patterns of missing data.

### Single-blinding

Our attempt at single-blinding was partially successful (6/25 early unblinding rate). This was achieved with relatively small effort on the part of the research, randomisation and clinical team. The effort on the part of respondents is unknown, but data collection was carefully designed to minimise this effort and maintain the quality of the respondent-interviewer relationship: all data collection prior to formal unblinding (all of t2 and first half of t3) was quantitative; formal unblinding occurred mid-t3 once all quantitative measures (including the primary outcome measure) other than service use data had been completed and before the qualitative interview; formal unblinding consisted of opening a sealed envelope in front of respondents, reading out the contents and asking respondents to confirm whether they had or had not received the service, it was therefore made clear that the researcher was now formally (and appropriately) unblinded and that they were free to report any contact with BIS or any other service in the subsequent 'use of services' questions and qualitative interview. Respondents were not therefore expected to maintain blinding whilst participating in qualitative interviews or answering questions about service use (of BIS or any other services). In the view of the experienced interviewer this strategy did not introduce distance between the respondent and interviewer, indeed respondents appeared to be encouraged at the 'scientific' nature of the study.

The source of the early unblinding was, in all cases, the respondents; this occurred most often at t2 when patients mentioned visits from named individuals. We recommend that the content of interviews at unblinded measurement points should be carefully considered when designing blinded-trial protocols: there is a need to find a balance between reducing the chances of accidental unblinding and the collection of data that is of value and that is blinded e.g. in the case of this study, primary outcome measure data was collected at each interview whilst the researcher was blinded but the collection of service use data and qualitative data was deferred until after the formal unblinding stage of the t3 interview.

### Baseline and outcome measures

The majority of measures tested in the trial proved suitable. Key exceptions were the WHO Performance Scale [16] and the Schedule for the Evaluation of Individual Quality of Life-Direct Weighting (SEIQoL-DW) [17]. The WHO Performance Scale lacked specificity for advanced COPD patients and will be replaced in Phase III by the Australia-modified Karnosfsky Performance Status Scale (AKPS) [36]. Based on the Karnofsky Performance Scale (KPS) [37] and the community care based Thorne-modified KPS (TKPS) [38], the AKPS accommodates any setting of care and is considered more relevant to palliative care [36].

Despite the high completion rate on the SEIQoL-DW, concerns about aspects of the process of administration led the research team to question the validity of the results obtained [39]. In Phase III quality of life will be assessed using a combination of the EQ-5D [40] (a brief measure of health status/health-related quality of life also required for the cost-effectiveness analyses), the full Chronic Respiratory Questionnaire [18] (Phase II used only the mastery scale), and qualitative interviews (open questions on the impact of breathlessness and the BIS on quality of life). This in line with the recommendations of Guthrie et al (2001) [41] and the 2005 MRC Clinical Trials Unit/Cicely Saunders Foundation 'Improving Research Methodology in Breathlessness' meeting [7].

### Role of Phase II

Grande & Todd (2000) describe the need for feasibility studies and careful piloting in order to carry out successful RCTs in palliative care [8]. Conducting this Phase II RCT provided crucial process and outcome data that has subsequently informed the robust design of Phase III: a single-blinded fast-track pragmatic RCT of BIS versus standard care for patients with any diagnosis. In addition, Phase II has provided additional data to further refine the service model of BIS prior to the commencement of Phase III.

This feasibility study focused on patients with COPD. It seems reasonable, due to similarities in disease trajectories, to expect its findings (in terms of the feasibility and acceptability of the methodology) to be generalisable to patients with other non-malignant conditions with similar trajectories (e.g. cystic fibrosis) with intractable breathlessness who may be referred to BIS in Phase III. However, generalisability to patients with breathlessness due to malignancies or those non-malignant illnesses that follow a malignant trajectory (e.g. interstitial lung disease) is unknown.

While none of the individual components of this study could be described as particularly novel (e.g. fast-track methodology, single-blinding, secondary care intervention delivered in the community, RCT in palliative care), the combination is. Von Gunten (2005) highlights the need for 'well-powered definitive studies of both existing and new approaches in terminally ill patients with the most common symptoms', including breathlessness [42]. Further, Bausewein et al (2007), reporting on an international meeting on breathlessness, noted the need for further examination of breathlessness intervention services in order to elucidate which components work most effectively in different conditions (e.g. malignant and non-malignant) [43]. Phase II has provided key data for the definitive Phase III RCT of BIS.

## Conclusion

In conclusion, this Phase II RCT has provided valuable information of the acceptability and feasibility of an RCT of BIS for patients with COPD. It has shown that: single-blinding is possible in a palliative care RCT and highlighted aspects of protocol design to reduce the unblinding rate; fast-track trials appear acceptable for patients with advanced COPD, their carers and referrers, as well as the clinicians (intervention providers); high response rates and low attrition can be achieved in palliative care RCTs with non-malignant patients; and, that baseline and outcome measures could be improved on. The trial has also provided vital data for the sample size calculations for Phase III (ClinicalTrials.gov NCT006-78405; NCRN Portfolio Study No. 4829).

## List of abbreviations

FEV1: forced expiratory volume in one second; NCRN: National Cancer Research Network.

## Competing interests

MF and IJH declare that they have no competing interests.

SB (founder of service) and PF were the clinicians providing the intervention (Breathlessness Intervention Service).

## Authors' contributions

MF co-designed Phase II, LREC and R&D approval, main researcher on Phase II, analysed feasibility data, authored first and subsequent drafts of paper. IJH co-applied for Phase II funding, co-designed Phase II, contributed to the revising the paper. PF co-designed intervention, intervention provider (clinician), contributed to later drafts of the paper. SB founded service and evaluation (Pre-clinical phase), co-designed intervention, intervention provider (clinician), co-applied for Phase II funding, co-designed Phase II, contributed to revising the paper. All authors read and approved the final draft.

## Pre-publication history

The pre-publication history for this paper can be accessed here:



## Supplementary Material

Additional file 1 **Table 7 – Quantitative patient and carer missing data for individual questionnaires/items for BIS Phase II RCT**. The data provided present quantitative patient and carer missing data for individual questionnaires and items. Click here for file
