# Supplementary material for: The feasibility of a single-blinded fast-track pragmatic randomised controlled trial of a complex intervention for breathlessness in advanced disease
Source: BMC Palliat Care. 2009 Jul 7;8:9. doi: 10.1186/1472-684X-8-9 (PMC2731082; doi:10.1186/1472-684X-8-9)
Supplement: Additional file 1 — Table 7 – Quantitative patient and carer missing data for individual questionnaires/items for BIS Phase II RCT. The data provided present quantitative patient and carer missing data for individual questionnaires and items. [file 1472-684X-8-9-S1.doc]

**Additional File 1:**

**Table 7: Quantitative patient and carer missing data for individual questionnaires / items for BIS Phase II RCT**

| Baseline / outcome measure | Missing data |  |  |  |  |  |  |  |  |  | Comments |
| --- | --- | --- | --- | --- | --- | --- | --- | --- | --- | --- | --- |
|  | **t1** |  | **t2** |  | **t3** |  | **t4** |  | **t5** |  |  |
|  | **Pt** | **Carer** | **Pt** | **Carer** | **Pt** | **Carer** | **Pt** | **Carer** | **Pt** | **Carer** |  |
| Demographics  (t1 only) | 0/13 | 0/12 | n/a | n/a | n/a | n/a | n/a | n/a | n/a | n/a | Date of birth missing for one carer, all remaining demographic data complete. |
| WHO Performance Scale [16] | 0/13 | n/a | 0/13 | n/a | 0/13 | n/a | 3/6 | n/a | 0/6 | n/a | Researcher completed. Researcher had difficulty deciding whether some patients Grade 3 or 4 e.g. P014 confined to the chair/bed all day except for trips to toilet which he managed himself, but most of his ‘self-care’ was done by a professional carer; a definition of ‘self-care’ may have helped. |
| Modified Borg Scale [19] | 0/13 | 0/12 | 0/13 | 1/12 | 0/13 | 0/12 | 0/6 | 2/6 | 0/6 | 0/6 | Many patients already familiar with the Modified Borg.  No missing administrations for patients; 3/48 administrations to carers missing. |
| VAS for ‘breathlessness’ and ‘distress due to breathlessness’ [7] | 0/13 | 1/12 | 0/13 | 2/12 | 0/13 | 0/12 | 1/6 | 1/6 | 0/6 | 0/6 | One patient administration missing at t4 (represents one out of 51 administrations of each VAS items).  One carer administration missing at t1 (incorrectly completed); two at t2 (one in correctly completed); one at t4 (4/48 potential administrations). |
| Mastery items from Chronic Respiratory Questionnaire [18] | 0/13 | n/a | 0/13 | n/a | 0/13 | n/a | 0/6 | n/a | 0/6 | n/a | No missing data. |
| Hospital Anxiety and Depression Scale [20] | 0/13 | 0/12 | 0/13 | 1/12 | 0/13 | 1/12 | 0/6 | 1/6 | 0/6 | 0/6 | Generally well received but some patients commented on or queried individual items in relation to their breathlessness. One patient referred to his ‘sleepiness’, rather than breathlessness, as affecting his choice of answers.  Only 2 items missing throughout the 714 items administered to patients (51 administrations x 14 items): both missing items were item 5 (‘Worrying thoughts go through my mind’) missed by the same respondent (WL group) at measurement points t3 and t5 (thus possible to impute values from the mean as per instructions).  Carers: two administrations missing (t2 and t4). At one t3 administration the last page was not completed (last 5 items). |
| SEIQoL-DW [17] | 0/13 | 0/12 | 1/13 | 2/12 | 0/13 | 1/12 | 3/6 | 4/6 | 0/6 | 0/6 | 4/51 potential patient and 7/48 potential carer administrations missing. One patient only able to identify three of the five required cues at t3. Despite high completion rate, concerns about administration questions the validity of the results obtained [38]. |
| Social Functioning | 0/13 | 1/12 | 1/13 | 1/12 | 0/13 | 0/12 | 1/6 | 1/6 | 0/6 | 0/6 | 2/51 potential patient and 2/48 potential carer administrations missing. |
| Service Use | 0/13 | 0/12 | 0/13 | 1/12 | 0/13 | 0/12 | 4/6 | 3/6 | 0/6 | 0/6 | No missing data for patients or carers at t1.  4/51 potential patient and 5/48 potential carer administrations missing. |
| Burden Interview [21] | n/a | 1/12 | n/a | 1/12 | n/a | 0/12 | n/a | 1/6 | n/a | 0/6 | 3/48 potential administrations missing including one partial administration at t1: items 7-12 (page 2 of questionnaire) missed. |
| Caregiver Appraisal Scale [22] | n/a | 0/12 | n/a | 3/12 | n/a | 0/12 | n/a | 1/6 | n/a | 0/6 | 4/48 potential carer administrations missing. |
